# Dual Face of Vγ9Vδ2-T Cells in Tumor Immunology: Anti- versus Pro-Tumoral Activities

**DOI:** 10.3389/fimmu.2017.01041

**Published:** 2017-08-28

**Authors:** Zheng Xiang, Wenwei Tu

**Affiliations:** ^1^Li Ka Shing Faculty of Medicine, Department of Paediatrics and Adolescent Medicine, Laboratory for Translational Immunology, University of Hong Kong, Hong Kong, Hong Kong

**Keywords:** Vγ9Vδ2-T cells, antitumor activity, pro-tumor activity, tumor immunity, tumor immunotherapy

## Abstract

Vγ9Vδ2-T cells are considered as potent effector cells for tumor immunotherapy through directly killing tumor cells and indirectly regulating other innate and adaptive immune cells to establish antitumoral immunity. The antitumoral activity of Vγ9Vδ2-T cells is governed by a complicated set of activating and inhibitory cell receptors. In addition, cytokine milieu in tumor microenvironment can also induce the pro-tumoral activities and functional plasticity of Vγ9Vδ2-T cells. Here, we review the anti- versus pro-tumoral activities of Vγ9Vδ2-T cells and discuss the mechanisms underlying the recognition, activation, differentiation and regulation of Vγ9Vδ2-T cells in tumor immunosurveillance. The comprehensive understanding of the dual face of Vγ9Vδ2-T cells in tumor immunology may improve the therapeutic efficacy and clinical outcomes of Vγ9Vδ2-T cell-based tumor immunotherapy.

## Introduction

Human γδ-T cells can be classified into two main subsets depending on the expression of T cell receptor (TCR) δ chain (1). Vδ1 γδ-T cells with different Vγ elements account for the majority of mucosal-associated lymphoid tissue γδ-T cells, and they mediate the immune responses to *Listeria monocytogenes*, Cytomegalovirus, and certain hematological malignancies (2, 3). In contrast, γδ-T cells bearing the Vδ2 gene with the co-expression of the Vγ9 chain (Vγ9Vδ2-T cells) are abundant in the peripheral blood and lymphoid organs of most healthy individuals, and they are involved in the first line of the immune responses to mycobacteria, Epstein–Barr virus (EBV), and some solid tumors (1, 2, 4–10).

The Vγ9Vδ2-T cell is an important component of immune effector cells that contribute to tumor immunosurveillance against many types of tumors, such as lymphoma, myeloma (11, 12), hepatocellular, and colorectal carcinoma (13), and prostate (14), lung (15), colon (16), breast (17), and ovary cancers (18). Recently, our group discovered a novel strategy to treat EBV-induced B-cell lymphoma by boosting Vγ9Vδ2-T cell immunity (19, 20). Vγ9Vδ2-T cells can directly kill tumor cells through the secretion of cytolytic molecules or indirectly prime and modulate immunological functions of other innate and adaptive immune cells to develop and establish profound antitumor immunity (21, 22). Unlike the conventional αβ-T cells, the Vγ9Vδ2-T cell is a member of the non-conventional lymphocyte family (23), and the antigen recognition of Vγ9Vδ2-T cells is major histocompatibility complex (MHC)-unrestricted (24, 25). Recent growing evidence has suggested that the Vγ9Vδ2-T cell is one of the most attractive candidates for antitumor immunotherapy. In this review, we will discuss recent advances in the basic Vγ9Vδ2-T cell research and evidence from clinical applications of Vγ9Vδ2-T cells. Most importantly, this review will provide an overview of the current knowledge about mechanisms of Vγ9Vδ2-T cell mediated antitumor immunity and the potential limitations of Vγ9Vδ2-T cell-based immunotherapy.

## Plasticity of Vγ9Vδ2-T Cells in Tumor Immunity

The antitumor activity of Vγ9Vδ2-T cells is influenced by their functional plasticity that is driven by environmental factors (26). Similar to αβ-T cells, Vγ9Vδ2-T cells also display the plasticity that contributes to their functional specialization (27). Accumulating evidence indicates that Vγ9Vδ2-T cells can differentiate into the cells with various characteristics associated with Th1-like, Th2-like, Th17-like, follicular T helper cells (Tfh)-like, or regulatory T cells (Treg)-like characteristics (26).

Upon phosphoantigen stimulation, Vγ9Vδ2-T cells preferentially differentiate into Th1-like cells with profound IFN-γ and TNF-α responses (28, 29). Th1-like Vγ9Vδ2-T cells can be induced through isopentenyl pyrophosphate (IPP) activation with IL-12 and anti-IL-4 antibody, and even the addition of IL-21 (30–32). Phosphoantigens and IL-2 can promote their cytolytic activity by upregulating CD56 expression and increasing granule secretion (32, 33). Interestingly, Vγ9Vδ2-T cells can also be polarized into Th2-like cells, which are characterized by increased secretion of IL-4 upon stimulation with IPP, IL-4, and anti-IL-12 antibody (30).

Vγ9Vδ2-T cells with Tfh-like functions can be induced by IL-21 and phosphoantigens stimulation (34, 35). These Tfh-like Vγ9Vδ2-T cells also have the capability to migrate into the lymph node germinal center (35). Similar to Tfh CD4^+^ T-cells, cell-to-cell contact is necessary for the B cell helper activity of the Tfh-like Vγ9Vδ2-T cells.

IL-17-producing γδ-T cells have been extensively discussed in the murine model (36). Recent findings also suggested that human γδ-T cells can produce IL-17 (37, 38). Some groups reported that naïve Vγ9Vδ2-T cells can be induced into the Th17-like phenotype or mixed Th1/Th17-like phenotype (39–41). Vγ9Vδ2-T cells require IL-1β, IL-23, and TGF-β, but not IL-6, for differentiation into Th17-like cells (41). In human colorectal cancer (CRC), activated inflammatory dendritic cells (DCs) polarize Vγ9Vδ2-T cells into IL-17-producing γδ-T cells, which can secrete high levels of IL-17 in an IL-23-dependent manner (42).

Upon stimulation of IPP in the presence of IL-15 and TGF-β, the Vγ9Vδ2-T cells can be induced into transcription factor forkhead box P3 (Foxp3)-expressing Treg-like γδ-T cells with regulatory/immunosuppressive function (43). When combined with IL-15, IL-2, TGF-β, and phosphoantigen stimulation, decitabine can also induce the immunoregulatory activity of Vγ9Vδ2-T cells (44).

Therefore, Vγ9Vδ2-T cells can be induced into different functional subsets depending on the cytokine milieu in the tumor microenvironment.

## Antitumoral Response of Vγ9Vδ2-T Cells

The antitumoral activity of Vγ9Vδ2-T cells has been well studied (45, 46). Their antitumoral activity mostly relies on the recognition of phosphoantigens and stressed molecules by TCR (47) and other cellular receptors (48), like NKG2D (49). These receptors can respond to perturbations in the endogenous isoprenoid biosynthesis and the presence of “danger signals” that occur during cell stress and malignant transformation. Other molecules can act as co-stimulatory signals to regulate the antitumoral activity of Vγ9Vδ2-T cells (48). Apart from the direct cytotoxicity of Vγ9Vδ2-T cells, these cells can also stimulate and regulate other immune components to establish the antitumoral activity (7, 50). Last, but not least, the homing receptors expressed on Vγ9Vδ2-T cells lead cell migration to tumor sites where they display broad and potent antitumoral activity (51).

### Mechanisms of Tumor Cell Recognition

#### TCRγδ Recognition

##### Non-Peptide Ligands

**Figure 1:**
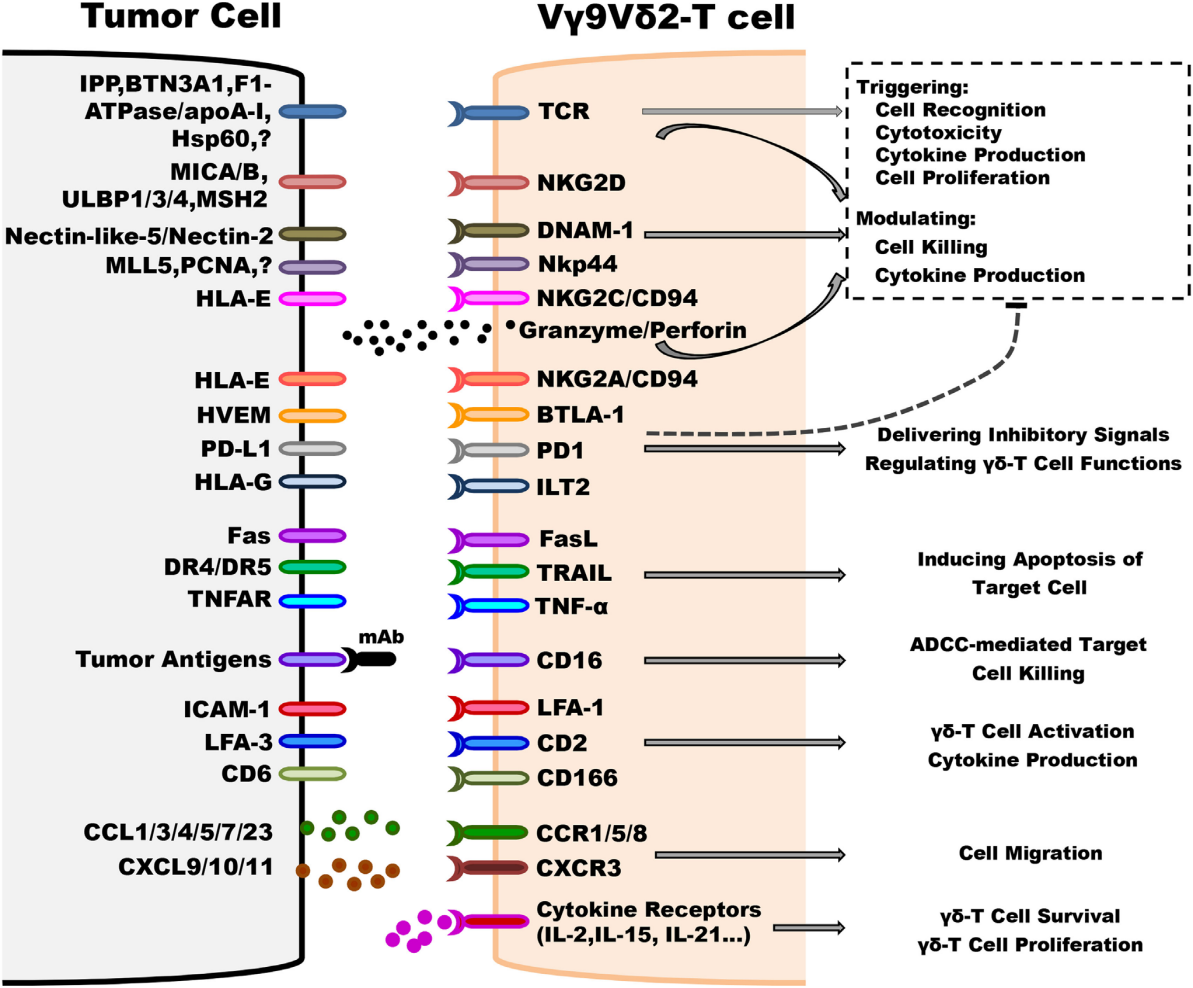
Underlying mechanisms implicated in regulating antitumoral activity of Vγ9Vδ2-T cells. Vγ9Vδ2-T cells can distinguish between tumorous and normal cells using T cell receptor (TCR) and other innate receptors to sense isopentenyl pyrophosphate (IPP) levels and stress signals (such as MICA/B, ULBP4, and MSH2) displayed on target cells. Most importantly, TCRγδ is the predominant factor that can trigger cell activation without any contribution of other co-stimulators, such as NKG2D. Following TCR-dependent activation, Vγ9Vδ2-T cells recognize and kill tumor cells by releasing effector molecules, such as granzymes and perforin, and Th-1 cytokines, inducing target cell apoptosis *via* Fas/FasL, TNF-related apoptosis-inducing ligand (TRAIL) and TNF-α pathways, and antibody-dependent cell-mediated cytotoxicity through CD16 expression. The activation threshold is finely regulated by inhibitory receptors, such as NKG2A/CD94. Moreover, adhesion patterns, such as lymphocyte function-associated antigen 1 (LFA-1)/intercellular adhesion molecule-1 (ICAM-1), are also involved in regulating the antitumoral activity of Vγ9Vδ2-T cells. The chemokine receptors, including CCR5, control the ability of Vγ9Vδ2-T cell to migrate to the tumor site. The survival and proliferation of Vγ9Vδ2-T cells are mostly modulated by different cytokines, such as IL-2 and IL-15.

In tumor immunosurveillance, although Vγ9Vδ2-T cells share some features with other immune cells, such as NK cells and αβ-T cells (21, 52), a distinctive characteristic of Vγ9Vδ2-T cells is the TCR-dependent recognition of non-peptidic phosphorylated antigens (also called phosphoantigens). Vγ9Vδ2-TCR recognizes the molecules that are normally expressed in specific conditions, such as when cells undergo stressful conditions. For example, (E)-4-Hydroxy-3-methyl-but-2-enyl pyrophosphate (HMBPP) and IPP are the products of the prokaryotic isoprenoid pathway and the mevalonate isoprenoid pathway, respectively (53–56). All of these products can be recognized by Vγ9Vδ2-T cells and lead to subsequent activation of Vγ9Vδ2-T cells. The levels of these naturally occurring metabolites are too low to be detected as a dangerous signal by Vγ9Vδ2-T cells in normal cells. The dysfunctional metabolism of malignant tumor cells can result in the accumulation of endogenous phosphoantigens that are recognized by Vγ9Vδ2-T cells. Furthermore, bisphosphonates (such as pamidronate and zoledronate) can cause tumor cells to be more sensitive to the cytotoxicity of Vγ9Vδ2-T cells *via* inhibition of the farnesyl pyrophosphate synthase enzyme in the isoprenoid pathway, which leads to IPP accumulation (57). Meanwhile, current findings have indicated that several molecules, such as F1-ATPase (combined with apolipoprotein A-I, called Apo A-I) (58, 59) and butyrophilin 3A1 (BTN3A1, CD277), might be involved with phosphoantigens to mediate Vγ9Vδ2-T cells activation (60, 61) (Figure 1).

##### Peptide Ligands

(1) Self ligands: in addition to non-peptide ligands, Vγ9Vδ2-T cells can also recognize some molecules of cellular origin, which could be capable of indicating cellular stress or malignant transformation (49, 62). Several self-antigens have been confirmed to bind to Vγ9Vδ2-TCR, including heat shock protein-60 (HSP 60) (63), U16-binding protein 4 (ULBP-4) (64), human MutS homolog 2 (hMSH2) (63, 65), and F1-ATP synthase (F1-ATPase) (59, 66). The expressions of these proteins are shown to be upregulated on the surface of different tumor cells and they can promote recognition by Vγ9Vδ2-T cells. It is intriguing that ULBP-4 and hMSH2 can also bind to NKG2D to induce the cytotoxicity of Vγ9Vδ2-T cells against tumor cells through TCR and NKG2D engagement (63–65) (Figure 1).
(2) Non-self ligands: tetanus toxoid (67), Ig λ light chain (68), and viral proteins, such as glycoprotein I from *Herpes simplex* (69) and staphylococcal enterotoxin A (70), are antigens that were reported to be capable of stimulating Vγ9Vδ2-T cell responses.

#### Cell Receptor Engagement

Besides the Vγ9Vδ2-TCR engagement, some other cellular receptors, especially the NK receptors (NKRs), are involved in the effective triggering of antitumoral responses of Vγ9Vδ2-T cells (49) (Figure 1). Together with previous studies, we reported that NKG2D can bind to its ligands (71), such as MICA, MICB, and ULBP-1, -2, -3, and -4, which are expressed in different tumors, including leukemia, lymphoma, ovarian, and colon carcinoma (72–74). In particular, the high expression level of ULBP1 indicates the susceptibility of lymphoma to Vγ9Vδ2-T cell-mediated cytolysis (74). Furthermore, ULBP-4 expression is detected on the cell surface of EBV-transformed lymphoid cells lines as well as on colon, ovarian, and liver cancer cells (64).

Another NKR implicated in tumor recognition by Vγ9Vδ2-T cells is the DNAX accessory molecule-1 (DNAM-1) (75, 76). Nectin-like-5 and Nectin-2, ligands of DNAM-1, are expressed on most hepatocellular carcinoma (HCC) cell lines (75). In addition, some Vγ9Vδ2-T cells also express NKp44, which can mediate their cytotoxic activity against multiple myeloma (MM) cell lines (77, 78).

Similar to NK cells, Vγ9Vδ2-T cells also express high levels of CD16 (FcγR III) upon phosphoantigen stimulation (79), and thus leading to antibody-dependent cell-mediated cytotoxicity (ADCC) against tumor cells (80–83).

## γδ-T Cells Act as Effector Cells

### Vγ9Vδ2-T Cells with Killer Functions

Interaction of TCR and/or NKG2D with their respective ligands can stimulate the activation of Vγ9Vδ2-T cells. Once activated, Vγ9Vδ2-T cells secrete IFN-γ and TNF-α, and increase the release of antitumor effector molecules, such as perforin and granzymes. The DNAM-1 signaling pathway can positively regulate the cytotoxic activity and IFN-γ secretion of Vγ9Vδ2-T cells against a broad range of tumors.

Antibody-dependent cell-mediated cytotoxicity mediated by Vγ9Vδ2-T cells can be activated *via* the binding of CD16 to antibodies, such as rituximab, trastuzumab, atumumab, and alemtuzumab, coated on the certain tumor cells (80–83).

In addition, activated Vγ9Vδ2-T cells can also induce tumor cell apoptosis *via* TNF-related apoptosis-inducing ligand and Fas/FasL pathways (84–86).

### Vγ9Vδ2-T Cells with Helper Functions

**Figure 2:**
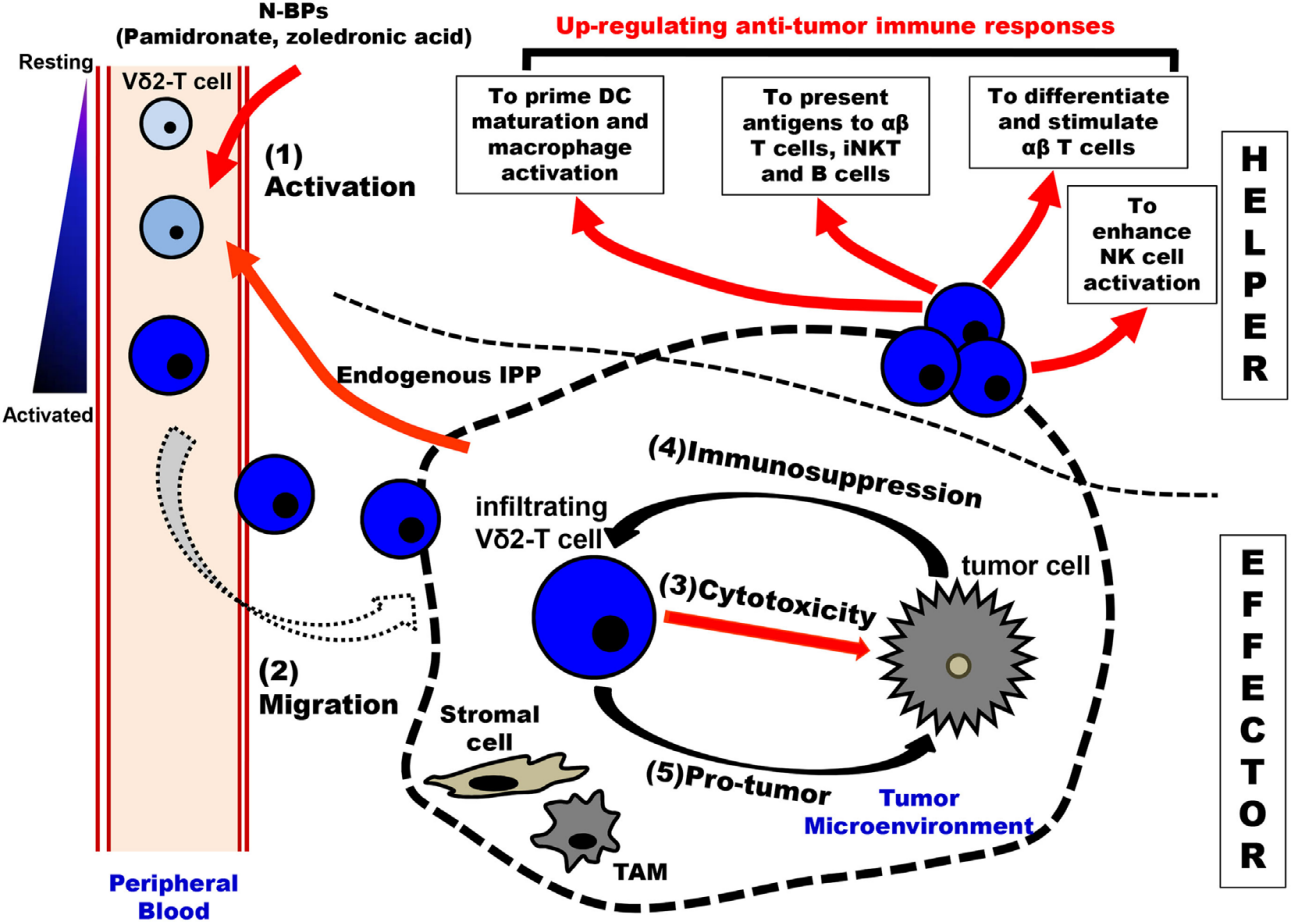
Schematic overview of immunological roles of Vγ9Vδ2-T cells in antitumoral surveillance. Vγ9Vδ2-T cells can make multiple contributions to antitumoral immunity, including “EFFECTOR” function and “HELPER” function. There are five steps involved in Vγ9Vδ2-T cell-associated effector functions: (1) Activation: upon stimulation by N-BPs (such as the administration of pamidronate) and increased-endogenous IPP produced by tumor cells, peripheral blood Vγ9Vδ2-T cells are activated in a T cell receptor-dependent manner. (2) Migration: the activated Vγ9Vδ2-T cells with an upregulated expression of chemokine receptors are recruited by gradient chemokines established by tumor cells. (3) Cytotoxicity: the infiltrating Vγ9Vδ2-T cells can recognize and kill tumor cells in the tumor site. (4) Immunosuppression: in the tumor microenvironment, tumor cells and other cells (like stromal cells and TAMs) produce immunosuppressive cytokines to induce the polarization of Vγ9Vδ2-T cells with Th-17 and Treg characteristics. Furthermore, the antitumoral activity of Vγ9Vδ2-T cells can be inhibited through the interaction of the inhibitory receptor and their corresponding ligands that are expressed by tumor cells and TAMs. (5) Pro-tumor: the polarized Vγ9Vδ2-T cells can secret IL-17 to support tumor growth directly. Moreover, IL-4, IL-10, IL-17, and TGF-β produced by the polarized Vγ9Vδ2-T cells not only recruit immunosuppressive MDSCs but also suppress innate and adaptive immune responses. Besides the “EFFECTOR” function, Vγ9Vδ2-T cells can also exhibit the “HELPER” functions in antitumor immune immunity. The activated Vγ9Vδ2-T cells can promote the maturation of DCs and the activation of macrophages. These Vγ9Vδ2-T cells can act as APC and initiate the differentiation and stimulation of tumor antigen-specific CD4^+^ T and CD8^+^ T cells. Moreover, Vγ9Vδ2-T cells can present antigens to iNKT and B cells. Vγ9Vδ2-T cells can promote NK cell antitumor response by delivering a costimulatory signal. APC, antigen presentation cell; DCs, dendritic cells; iNKT, invariant NKT cells; IPP, isopentenyl pyrophosphate; MDSCs, myeloid-derived suppressor cells; NK, natural killer cell; N-BPs, nitrogen-containing bisphosphonates; and TAMs, tumor-associated macrophages.

Activated Vγ9Vδ2-T cells may secrete chemokines, such as C-C motif chemokine ligand 3 (CCL3), CCL4, C-X-C motif chemokine 10 (CXCL10), and CXCL13, to recruit αβ-T cells, B cells, NK cells, and macrophages/DCs to the tumor site (22, 31, 87). Activated Vγ9Vδ2-T cells not only stimulate DC maturation and macrophage activation (88, 89), but also induce CD4^+^ and CD8^+^ αβ-T cell differentiation for enhancing antitumoral activity through secretion of IFN-γ and TNF-α and upregulation of CD40L expression (88, 90, 91). Moreover, activated Vγ9Vδ2-T cells can mimic antigen presentation cell (APC) functions by stimulating the antitumoral activity of αβ-T cells through the upregulation of several surface molecules, such as MHC I and II, CD40, CD83, and CD86 (92, 93). Activated Vγ9Vδ2-T cells can also present glycolipid antigens to iNKT cells through the uptake of CD-1d by trogocytosis. Subsequently, activated iNKT cells can trigger downstream reactions to boost antitumoral immunity (94). Furthermore, activated CD137L^+^ Vγ9Vδ2-T cells can stimulate the antitumoral activity of NK cells by acting as a co-stimulator through interacting with CD137 expressed on NK cells. Thus, the costimulatory signals can upregulate the cytotoxic activity of NK cells to kill the solid tumor cells, which usually show resistance to NK cells (95). Although the contribution of IL-17 in tumor surveillance is still controversial (96–99), some *in vitro* and *in vivo* evidence have indicated that IL-17 secreted by Vγ9Vδ2-T cells might be involved in antitumoral immune responses *via* indirect mechanisms (96) (Figure 2).

### Vγ9Vδ2-T Cells with Homing Functions

CC and CXC chemokines are produced by most tumor cells, such as breast, cervix, pancreatic, and ovarian tumor cells (57). To provide optimal protection against tumor cells, the cytotoxic Vγ9Vδ2-T cells must migrate from the bloodstream to tumor site (33, 100, 101) (Figure 2). Unlike Vδ1 γδ-T cells which express lymph node homing chemokine receptor C-C chemokine receptor 7 (CCR7), the circulating Vγ9Vδ2-T cells preferentially express inflammatory homing chemokine receptor CCR5, which can mediate the migration of Vγ9Vδ2-T cells to CCR5 ligands that are expressed in tumor cells (20, 102). Apart from the predominantly expressed CCR5, Vγ9Vδ2-T cells also express other chemokine receptors including CCR1, CCR8, and C-X-C motif chemokine receptor 3, which are involved in regulating the homing ability of Vγ9Vδ2-T cells (13, 87, 103).

In addition, some of the adhesion molecules, such as lymphocyte function-associated antigen 1 (LFA-1), L-selectin, and CD44v6, are also involved in the migration of Vγ9Vδ2-T cells to the tumor site (104) (Figure 1).

## Regulation Mechanisms of γδ-T Cell Activation

### Vγ9Vδ2-T Cell Activation

In tumor immunosurveillance, the antitumoral activities of Vγ9Vδ2-T cells is composed of several steps (23): (1) detecting and sensing any types of stress signals in a non-MHC-restricted manner; (2) producing a huge quantity of effector molecules that can kill tumor cells through direct and indirect mechanisms; and (3) exhibiting potent cytotoxic and cytolytic activities against a broad panel of tumors. In order to tightly monitor the initiation and development of tumorigenesis, the activation of Vγ9Vδ2-T cells is triggered by TCR-mediated recognition and precisely regulated by various innate immune cells and cytokine receptors. Furthermore, the activation and differentiation of Vγ9Vδ2-T cells are molecularly controlled by surface receptors that capture key extracellular cues and convey downstream intracellular signals (105). More detailed information can be found in Figure 1.

### Regulation of Vγ9Vδ2-T Cell Activation

#### Regulation by TCR

Vγ9Vδ2-T cells mainly recognize the non-peptide ligands, phosphoantigens, which are shown to act as a trigger signal to activate Vγ9Vδ2-T cells (105). Cipriani et al. concluded that IPP induces rapid and persistent PKC-dependent phosphorylation of ERK 1/2, p38 MAPK, and JNK, which can lead to the activation of NF-κB and AP-1 and the secretion of IFN-γ and TNF-α (106). Some studies have shown that crosslinking of CD3 and phosphoantigens stimulation can induce highly sustained calcium signaling in Vγ9Vδ2-T cells *via* phosphorylation of Zap70, Pi3K, LAT, ERK 1/2, and p38 MAPK (105, 107, 108).

Due to upregulated self-antigen expressions in the transformed tumor cells, Vγ9Vδ2-T cells discriminate these malignant cells by directly binding to these self-antigens (49), such as HSP 60, ULBP-4, hMSH2, and F1-ATPase, which can enhance the activation and cytotoxicity of Vγ9Vδ2-T cells.

#### Regulation by NK-Associated Receptors

The expression of NK-associated receptors is a distinguishing feature of Vγ9Vδ2-T cells that mediates the recognition of stress ligands expressed by normal cells during infection and cell transformation by different pathogens. NKG2D and its ligands, such as MICA/B and ULBPs (64, 74, 109), are well-defined NK-associated receptors and ligands for Vγ9Vδ2-T cells. For example, expressions of these ligands are normally induced during cellular stress, such as DNA damage that occurs in tumor cells.

Whether NKG2D plays a primary stimulatory or a co-stimulatory role in Vγ9Vδ2-T cells is still being debated (49). Some evidence have indicated that an additive effect of the NKG2D pathway on TCR-mediated activation through increasing cytokine production (110), and upregulating intracellular calcium mobilization and enhancing cytotoxic activity (105). On the other hand, other studies have reported that the NKG2D pathway alone can activate Vγ9Vδ2-T cells without TCR engagement (111), and the blockade of the NKG2D pathway, but not the blockade of γδ-TCR, can inhibit the cytotoxicity of Vγ9Vδ2-T cells against some hematological tumors (74).

DNAX accessory molecule-1 is another NK-associated receptor that regulates Vγ9Vδ2-T cells, whereby blocking the interaction between DNAM-1 and its ligands, such as Nectin-like-5 and Nectin-2, could impair the cytotoxic capacity and IFN-γ production of Vγ9Vδ2-T cells against HCC cells (75).

A recent study demonstrated that NKp44, a natural cytotoxicity receptor, is involved in the cytotoxicity of Vγ9Vδ2-T cells against MM cell lines that lack the expression of NKG2D ligands (77). Because Vγ9Vδ2-T cells are potentially highly self-reactive, TCR-mediated activity needs to be tightly controlled by a close interplay between activating and inhibitory NKRs (105). Like NK cells, most human circulating Vγ9Vδ2-T cells express several inhibitory NKRs belonging to the lectin-like receptor family (such as the NKG2A/CD94 heterodimer), or the immunoglobulin (Ig) family [such as Ig-like transcript 2 (ILT-2)]. Upon interaction with the classical and/or non-classical MHC I molecules, the inhibitory signals are delivered by these receptors to Vγ9Vδ2-T cells. This mechanism allows TCR-activated Vγ9Vδ2-T cells to target the tumor cells rather than the normal cells depending on the expression level of the MHC I molecules, and thus preventing self-reactivity while enhancing antitumoral activity (105). In addition, NKG2C/CD94 also can regulate Vγ9Vδ2-T cell effector functions, including cytokine secretion, cell proliferation, and cytotoxic activity (112).

#### Regulation by Co-Stimulatory Receptors

There are two main types of costimulatory receptors that are expressed on Vγ9Vδ2-T cells: the Ig and tumor necrosis factor receptor (TNFR) superfamilies. Ribot et al. suggested that CD28, which is an important member of the Ig superfamily, is constitutively expressed on lymphoid Vγ9Vδ2-T cells. Co-stimulation with CD28 promotes the survival and proliferation of Vγ9Vδ2-T cells by enhancing IL-2 production, whereas blocking antibodies target its B7 ligands (CD80 and CD86) can inhibit cell survival and proliferation (113). CD27 is a member of the TNFR superfamily that is expressed in 80% of Vγ9Vδ2-T cells (114). CD27 pathway has been confirmed to play critical roles in co-stimulating Vγ9Vδ2-T cell activation. Furthermore, CD27 is also involved in promoting cell proliferation, upregulating the antiapoptotic gene *Bcl2a1* to maintain cell survival and enhancing IFN-γ production and cytotoxicity of Vγ9Vδ2-T cells (105, 114). Upon activation, CD30, another member of the TNFR superfamily, is also expressed on Vγ9Vδ2-T cells and it can increase pro-inflammatory cytokine production and promote TCR-induced calcium fluxes (115). A high level of CD137L that is expressed on activated Vγ9Vδ2-T cells can transmit a reversal signal to regulate Vγ9Vδ2-T cell activation (95, 116).

#### Regulation by Adhesion Molecules

In addition to TCR and NKR, other surface receptors, such as adhesion molecules, are also important for regulating Vγ9Vδ2-T cell function. To date, several adhesion molecules, including LFA-1/intercellular adhesion molecule-1 (ICAM-1) (117), CD2/LFA-1/3 (118), and CD6/CD166 (119), have been identified as regulators in modulating Vγ9Vδ2-T cell activation.

High expressions of LFA-1 and its ligand, ICAM-1, have been detected on the surface of Vγ9Vδ2-T cells and most of the tumor cell lines, respectively. LFA-1/ICAM-1 adhesive interaction is necessary but not sufficient for IFN-γ production and the cytotoxicity of Vγ9Vδ2-T cells (117). The interaction between LFA-3 on Vγ9Vδ2-T cells and CD2 on lymphoid cells can stimulate TNF-α secretion by Vγ9Vδ2-T cells (118). Additionally, CD6 is a member of the scavenger receptor family, which is also expressed on the surface of Vγ9Vδ2-T cells. CD6 binds to its ligand, CD166, which is associated with the capability of tumor cells to activate Vγ9Vδ2-T cells upon phosphoantigen induction (119).

Most importantly, the interactions between LFA-1/ICAM-1 and CD2/LFA-3 can stabilize the immunological synapses after Vγ9Vδ2-TCR/phosphoantigen triggers the formation of immunological synapses (120, 121).

#### Regulation by Toll-Like Receptors (TLRs)

The roles of TLRs in regulating the activation of Vγ9Vδ2-T cells are not completely understood. After ligation of TLR2, TLR3, and TLR5, the Vγ9Vδ2-T cells can produce IFN-γ, TNF-α, granulocyte-macrophage colony-stimulating factor, CCL3, and CCL5 (122). The triggering of TLR3, 4, 5, and 9 can also induce the early activation of Vγ9Vδ2-T cell and the production of IFN-γ (105). In addition, TLR3 and TLR7 agonists can enhance the antitumoral activity of Vγ9Vδ2-T cells against adenocarcinoma cells (123). Nevertheless, these responses require simultaneous stimulation of Vγ9Vδ2-TCR (105).

#### Regulation by Cytokine Receptors

The interleukin receptors are essential for the development and homeostasis of Vγ9Vδ2-T cells due to the pivotal effect of interleukins on cell proliferation, differentiation, and survival, and the regulation of the immunological functions of the Vγ9Vδ2-T cells. IL-2 and IL-15 can be used to stimulate Vγ9Vδ2-T cell expansion, but they cannot fully induce effector functions without γδ-TCR-mediated activation and downstream signals, such as the ERK and AKT pathway (124, 125). IL-12 and IL-18 are reported to be beneficial for differentiation into IFN-γ^+^ Vγ9Vδ2-T cells (126). Recent studies also indicated that other interleukins, such as IL-23, IL-1β (39, 40, 127), and IL-21 (34, 35), are also involved in the induction of Vγ9Vδ2-T cell plasticity.

#### Regulation by Inhibitory Receptors

Like inhibitory NKRs, some other inhibitory molecules, such as programmed cell death protein-1 (PD-1) and B- and T-lymphocyte attenuator (BTLA), act in a similar manner in Vγ9Vδ2-T cells. PD-1 and BTLA can negatively regulate Vγ9Vδ2-T cell responses. Vγ9Vδ2-T cells do not normally express detectable PD-1; however, once activated, the expression of PD-1 is upregulated in Vγ9Vδ2-T cells (128). Iwasaki et al. found that the interaction of PD-1 and PD-L1 can attenuate the antitumoral activity of Vγ9Vδ2-T cells by downregulating IFN-γ production and lowering cytotoxicity (128). BTLA engagement can downregulate phosphoantigen/γδ-TCR-mediated signaling and inhibit Vγ9Vδ2-T cell proliferation, including the response to lymphoma cells (129). Moreover, the HLA-G expressed on the surface of tumor cells can bind to the ILT-2 on Vγ9Vδ2-T cells. This interaction could impair cytokine production and the cytotoxicity of Vγ9Vδ2-T cells (130).

## Pro-Tumoral Response of Vγ9Vδ2-T Cells

**Figure 3:**
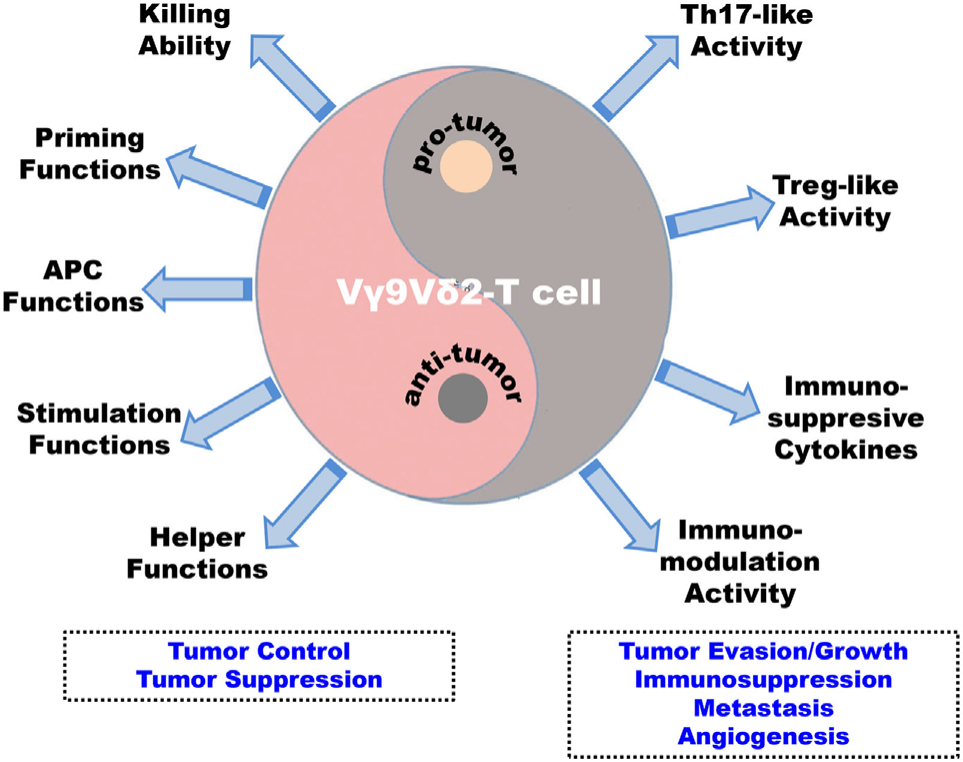
Antitumoral versus pro-tumoral activities of human Vγ9Vδ2-T cells. Vγ9Vδ2-T cells display dual effects in the immune surveillance against tumors, including antitumoral and pro-tumoral functions. On one hand, Vγ9Vδ2-T cells can control and suppress tumor development directly through potent cytotoxic capability and indirectly by regulating other innate and adaptive immune cells to establish antitumoral immune responses. First, Vγ9Vδ2-T cells can recognize and eliminate tumor cells with their potent killing ability. Second, they can promote dendritic cell maturation and macrophage activation. Third, Vγ9Vδ2-T cells can present antigens to αβ-T cells and iNKT cells. Fourth, they can stimulate and enhance NK cell antitumoral response. Finally, Vγ9Vδ2-T cells can help B cells produce antibodies. On the other hand, in the tumor microenvironment, the Vγ9Vδ2-T cells can be induced into Th-17-like cells and Treg-like cells by specific stimuli and they can secret immunosuppressive cytokines, such as IL-17, IL-10, and TGF-β, to suppress and modulate innate and adaptive antitumoral immunity. These strategies will be beneficial for tumor evasion/growth, metastasis, and angiogenesis.

Recently, more evidence indicates that Vγ9Vδ2-T cells can display the functions for promoting tumor development through direct or indirect strategies (27). As mentioned above, the differentiation of unique subpopulations of Vγ9Vδ2-T cells with immunosuppressive features can be induced in the presence of specific stimuli, such as in the tumor-established microenvironment (Figure 2). For example, Vγ9Vδ2-T cells may display Th2-, Th17-, or Treg-like profile and produce IL-4, IL-17, IL-10, and TGF-β, respectively. IL-10 and TGF-β are the cytokines with immunosuppressive activity, while IL-10 can impair APC function of DCs, and induce the pro-tumoral functions of Vγ9Vδ2-T cells (131, 132). Some evidence indicates that IL-17 produced by Th17-like Vγ9Vδ2-T cells can directly promote the proliferation and dissemination of tumor cells in breast cancer (133). Most importantly, in the tumor microenvironment, IL-17 plays a critical regulatory role to other immune components, such as MDSCs and macrophage, and thus indirectly influences tumor immunosurveillance (42). Moreover, similar to classical Treg cells, the suppressive effect of regulatory-like Vγ9Vδ2-T cells also depends on cell-to-cell contact *via* CD86/CTLA-4 and PD-L1/PD-1 interactions between activated Vγ9Vδ2-T cells and their responder cells (134) (Figure 3).

More recently, Peters et al. demonstrated that Vγ9Vδ2-T cell is a major source of IL-9 *in vitro* (135). According to this study, Vγ9Vδ2-T cells can secret IL-9 when culturing with TGF-β and IL-15 and in the absence of IL-4. IL-9-producing CD8^+^ T cells and Th9 cells are always considered as new players in antitumoral immune responses (136–138); however, in some studies, IL-9 also displays potential pro-tumoral activity. Qiu et al. found that in ALK^+^ anaplastic large-cell lymphoma, IL-9 can promote lymphoma cell proliferation and colony formation by regulating Jak3 activation in an autocrine manner (139). Furthermore, IL-9 can abolish the establishment of immunological memory to tumor re-challenge and exhibits an immunomodulatory role in pro-tumoral immunity (140). Indeed, elevated serum IL-9 was found in the patients with metastatic breast cancer or diffuse large B-cell lymphoma (DLBCL) (141, 142). Meantime, Lv et al. found that IL-9 can support the survival of DLBCL cells and enhance the resistance of these tumor cells to chemotherapeutic drugs by upregulating p21CIP1 genes (141). Thus, in tumor immunity, the exact immunological roles of IL-9-producing Vγ9Vδ2-T cells are still unknown. So far, the evidence for pro-tumoral activity of Vγ9Vδ2-T cells mostly comes from *ex vivo* experiments. More clinical evidence is needed to evaluate the potential pro-tumor ability of Vγ9Vδ2-T cells *in vivo*.

## Therapeutic Effects of Vγ9Vδ2-T Cells in Clinical Trials

To date, two main therapeutic strategies based on Vγ9Vδ2-T cells have been proposed for tumor immunotherapy: the *in vivo* expansion of Vγ9Vδ2-T cells by aminobisphosphonates, and the adoptive transfer of *ex vivo*-expanded Vγ9Vδ2-T cells (143). Several clinical trials have been initiated to evaluate the safety and therapeutic efficacy of Vγ9Vδ2-T cell immunotherapy based on *in vivo* and *ex vivo* expansion. The clinical outcomes of these strategies have been confirmed in patients with different types of tumors, including leukemia/lymphoma, melanoma, RCC, hormone-refractory prostate cancer, breast cancer, NSCLC, CRC, MM, gastrointestinal tumors, ovarian cancer, cervical cancer, and bone cancer (45, 57). For instance, Wilhelm et al. first reported that administration of pamidronate and low-dose of IL-2 to expand Vγ9Vδ2-T cells *in vivo* was well tolerated and could induce objective tumor response in patients with low-grade non-Hodgkin lymphoma or MM (18). Lang and coworkers conducted a pilot trial to determine the therapeutic effects of zoledronate with low-dose of IL-2 in patients with RCC (144). Furthermore, zoledronate with low dose of IL-2 have also been tested in treating prostate cancer and advanced breast cancer where partial remissions haves been reported (14, 17). Bennouna et al. conducted a phase I study that, after treatment of bromohydrin pyrophosphate (BrHPP) and IL-2, in total of 28 patients, 12 patients had stable disease and 16 had progressive disease after three cycles of administration (145).

Adoptive transfer of zoledronate-expanded Vγ9Vδ2-T cells was reported to induce a complete remission of RCC patient with lung metastasis. (146). Kobayashi et al. also reported that adoptive transfer of Vγ9Vδ2-T cells in combination with zoledronate and IL-2 was well tolerated and the objective clinical responses could be achieved in some patients with advanced RCC (147). More detailed information about clinical studies of Vγ9Vδ2-T cells had been systematically reviewed in recent articles summarized by Deniger et al. and Kobayashi and Tanaka (45, 148).

Although these two strategies have yielded clinical success, there are still some limitations. For the *in vivo* expansion and activation of Vγ9Vδ2-T cells, one of the limitations is that the sustained proliferative activity is impaired, probably due to an energy or exhaustion of Vγ9Vδ2-T cells induced by the successive infusions of BrHPP and IL-2 (45, 149). Additionally, Kalyan et al. demonstrated that neutrophils in human peripheral blood could uptake of zoledronate and cause the suppression of Vγ9Vδ2-T cells (150). Fowler et al. suggested that systemic use of zoledronate reduced the tumor homing ability of Vγ9Vδ2-T cells (151). Indeed, repeated administration of zoledronate and IL-2 in the patients with breast cancer and RCC could inhibit the proliferative capacity of Vγ9Vδ2-T cells and reduced the responsiveness of Vγ9Vδ2-T cells to re-stimulation (144, 152). For the adoptive cell transfer therapy, the main problem is difficulty to expand Vγ9Vδ2-T cells *ex vivo* from the advanced cancer patients with limited initial number of Vγ9Vδ2-T cells, especially after radiotherapy and chemotherapy (45). Moreover, the effect of immunosuppressive tumor microenvironment on adoptively transferred Vγ9Vδ2-T cells is still not clear.

Up to now, aminobisphosphonates have only been found to target and stimulate Vγ9Vδ2-T cell subsets, but they do not target other subpopulations of γδ-T cells, such as Vδ1-T cells. Some synthetic phosphoantigens, such as BrHPP, HMBPP, and 2-methyl-3-butenyl-1-pyrophosphate (2M3B1PP), are also found to activate and expand Vγ9Vδ2-T cells by mimicking the effects of aminobisphosphonates (153). Upon stimulation by these compounds, Vγ9Vδ2-T cells can enhance their antitumoral activities by release of IFN-γ and TNF-α (57). The administration of BrHPP was also tested in a Phase I study; the data showed that the BrHPP treatment was well tolerated and expansion of Vγ9Vδ2-T cells was successfully induced in patients with solid tumors (145). In addition to synthetic phosphoantigens, some monoclonal antibodies targeting γδ-TCR are also good candidates for tumor immunotherapy. Starick et al. demonstrated that BTN3A (CD277)-specific monoclonal antibody 20.1 induced TCR-mediated activation of Vγ9Vδ2-T cells (154). This study not only provided the novel mechanisms involved in Vγ9Vδ2-T cell activation but also showed the potential for using BTN3A-specific antibodies to manipulate Vγ9Vδ2-T cell immunity in tumor immunotherapy (154, 155).

Some recent studies have focused on the development of novel protocols to expand Vγ9Vδ2-T cells using immobilized antigens (156), agonistic monoclonal antibodies (15, 101), and tumor-driven artificial antigen-presenting cells (157, 158). Meanwhile, Vγ9Vδ2-T cells are also amenable to genetic modifications *via* the introduction of αβ TCRs (159), and chimeric antigen receptors (160). Furthermore, Vγ9Vδ2-T cells are suggested to act as a novel cellular vaccine to treat cancer patients (161).

## Conclusion

Recent advances in Vγ9Vδ2-T cell research have paved a way for developing innovative therapeutic strategies for tumor immunotherapy. As discussed in this review, Vγ9Vδ2-T cells can recognize tumor cells through TCR and other cell surface receptors, and their antitumoral activity is strictly regulated by activating and inhibitory receptors and their ligands. In addition, stimuli/cytokine milieu in tumor microenvironment can also induce pro-tumoral activity and functional plasticity of Vγ9Vδ2-T cells. Further study on the dual face of Vγ9Vδ2-T cells in tumor immunology could optimize current therapeutic protocols and improve the therapeutic efficacy and clinical outcomes of Vγ9Vδ2-T cell-based tumor immunotherapy.

## Author Contributions

ZX and WT wrote the manuscript. WT revised the manuscript.

## Conflict of Interest Statement

The authors declare that the research was conducted in the absence of any commercial or financial relationships that could be construed as a potential conflict of interest.
